# Finding new *Arabidopsis* receptor kinases that regulate compatible pollen-pistil interactions

**DOI:** 10.3389/fpls.2022.1022684

**Published:** 2022-09-15

**Authors:** Stephen J. Bordeleau, Laura E. Canales Sanchez, Daphne R. Goring

**Affiliations:** ^1^ Department of Cell and Systems Biology, University of Toronto, Toronto, ON, Canada; ^2^ Centre for the Analysis of Genome Evolution and Function, University of Toronto, Toronto, ON, Canada

**Keywords:** *Arabidopsis*, pollen-pistil interactions, pollen tube, receptor kinases, ligands

## Abstract

Successful fertilization of a flowering plant requires tightly controlled cell-to-cell communication between the male pollen grain and the female pistil. Throughout *Arabidopsis* pollen-pistil interactions, ligand-receptor kinase signaling is utilized to mediate various checkpoints to promote compatible interactions. In *Arabidopsis*, the later stages of pollen tube growth, ovular guidance and reception in the pistil have been intensively studied, and thus the receptor kinases and the respective ligands in these stages are quite well understood. However, the components of the earlier stages, responsible for recognizing compatible pollen grains and pollen tubes in the upper reproductive tract are less clear. Recently, predicted receptor kinases have been implicated in the initial stages of regulating pollen hydration and supporting pollen tube growth through the upper regions of the reproductive tract in the pistil. The discovery of these additional signaling proteins at the earlier stages of pollen-pistil interactions has further elucidated the mechanisms that *Arabidopsis* employs to support compatible pollen. Despite these advances, many questions remain regarding their specific functions. Here, we review the roles of the different receptor kinases, integrate their proposed functions into a model covering all stages of pollen-pistil interactions, and discuss what remains elusive with regard to their functions, respective binding partners and signaling pathways.

## Introduction

**Figure 1:**
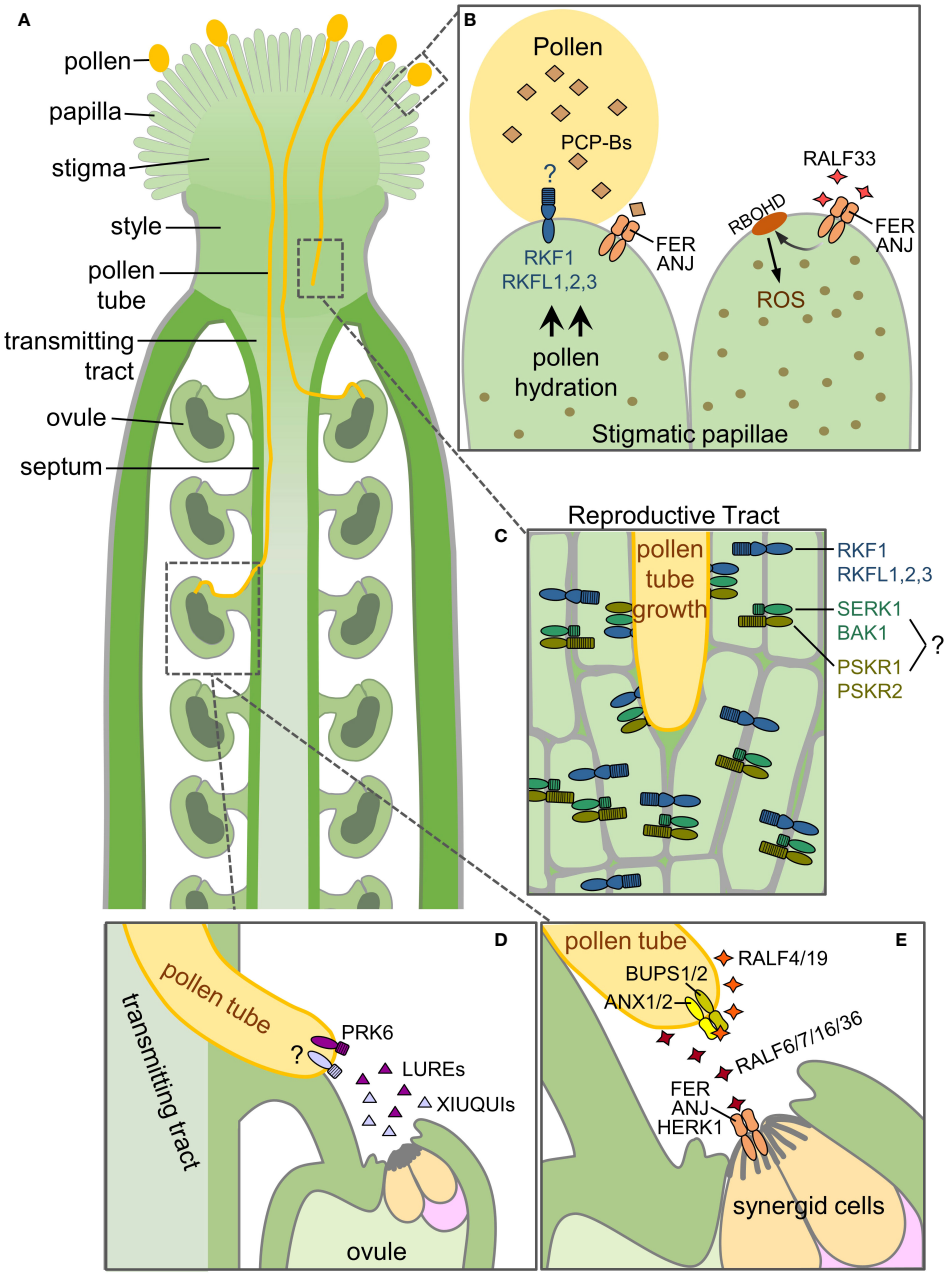
Receptor kinases and their respective ligands for compatible pollen-pistil interactions. This diagram illustrates the RLKs, RKs and their ligands functioning at different stages of the pollen-pistil interactions in *Arabidopsis*. **(A)** Overview of the different structures in a pollinated pistil. **(B)** Pollen reception at the stigmatic papilla. **(C)** Growth of the pollen tube through the reproductive tract (stigma, style, transmitting tissue). **(D)** Ovular guidance of the pollen tube to an unfertilized ovule. **(E)** Pollen tube reception at the ovule. See text for further details.

The process leading to fertilization in flowering plants begins with the deposition of pollen, the male gametophyte, on the stigma, the receptive part of the female pistil. A general property of Brassicaceae pistils, including *Arabidopsis*, is the presence of dry stigmas which possess cellular signalling pathways for selective pollen hydration (reviewed in Abhinandan et al., 2022). Brassicaceae pollen grains carry a pollen coat on the surface with a number of small Pollen Coat Proteins (PCPs) that can serve as signalling peptides (Doughty et al., 1993; Wang et al., 2017). In *Arabidopsis*, the recognition of compatible pollen grains involves receptor kinases (RKs) on the surface stigmatic papillae and leads to the papillae releasing water for pollen hydration. Hydration of the desiccated pollen grains is a critical step as it is required to initiate pollen metabolism and germination of a pollen tube. In contrast, pistils with wet stigmas such as tomato (*Solanum lycopersicum*) have little control over pollen hydration, and RKs begin to regulate these interactions during pollen tube growth (Huang et al., 2014; reviewed in Dickinson, 1995; Kim et al., 2021). In *Arabidopsis*, once a pollen tube emerges, it grows down to the base of the stigmatic papilla where it continues to grow through the stigma and style to reach the transmitting tract. Using RK-mediated autocrine and paracrine signalling, the pollen tube will grow down the transmitting tract towards an unfertilized ovule where it emerges from the septum and grows along the funiculus of the ovule to enter the micropyle, ceasing growth as it reaches a receptive synergid cell (**Figure 1A**). The pollen tube then bursts to release the two sperm cells which fuse to the egg cell and central cell culminating in double fertilization. All these steps depend on tightly controlled cell-to-cell communication between male and female tissues to deliver the male gametophytes to an ovule (reviewed in Johnson et al., 2019; Hafidh and Honys, 2021; Kim et al., 2021; Robichaux and Wallace, 2021)

As is common with flowering plants, *Arabidopsis* has a large family of receptor-like kinase (RLK) genes, and an increasing number of these RLKs have been shown to encode true RKs with the associated ligand(s) identified; thus, serving as primary sensors in the perception of extracellular signals (reviewed in Escocard de Azevedo Manhaes et al., 2021). The predicted RLKs have been further subdivided in several different subgroups based on the type of domains identified in their predicted extracellular domains (Shiu and Bleecker, 2001; reviewed in Dievart et al., 2020). In the context of *Arabidopsis* reproduction, members of the *Catharanthus roseus* Receptor-Like Kinase 1-like (CrRLK1L) and the Leucine-Rich Repeat (LRR) RLK subfamilies have proven to be critical regulators, and they are involved at every stage of this complex, multifaceted process (reviewed in Hafidh and Honys, 2021; Kim et al., 2021). Much of these discoveries have centered on the later stages where receptor kinases and signaling events guide pollen tubes to unfertilized ovules, regulate pollen tube reception at the synergid cells, and prevent multiple pollen tubes from approaching an unfertilized ovule. More recently, additional RKs and RLKs have been discovered in the pistil that act at earlier stages of pollen-stigma interactions and pollen tube growth through the upper regions of the reproductive tract (Stuhrwohldt et al., 2015; Lee and Goring, 2021; Liu et al., 2021a). This review will integrate these recent discoveries to provide some context for where they function in compatible pollen-pistil interactions and highlight what remains elusive in terms of their proposed functions.

## Negative and positive regulators at the pollen-stigma interface

In the absence of pollen, the stigma has been found to have high levels of reactive oxygen species (ROS) (McInnis et al., 2006; Zafra et al., 2016). Recently, high stigmatic ROS levels in *Arabidopsis* have been proposed to have an inhibitory effect on pollen hydration. A complex of two CrRLK1L members, ANJEA (ANJ) and FERONIA (FER), are proposed to establish an autocrine signalling cascade to maintain these ROS levels in *Arabidopsis* sigmatic papillae in the absence of pollen (Liu et al., 2021a). A connection between FER and a ROS pathway had been previously established in the context of root hair elongation (Duan et al., 2010) and pollen tube reception (described below; Duan et al., 2014). The extracellular domains of CrRLK1L family members contain two malectin-like domains which have been previously found to bind to secreted cysteine-rich peptides known as RAPID ALKALINIZATION FACTORs (RALFs) (reviewed in Galindo-Trigo et al., 2020b). In the context of the stigma, RALF33 which is proposed to be secreted by the stigma was shown to bind ANJ/FER. RALF33 perception by the stigmatic ANJ/FER complex then leads to the induction of a RAC/ROP-NADPH oxidase (RBOHD) pathway for ROS production. As the high ROS state is inhibitory to pollen hydration, it would need to be reversed for compatible pollen hydration. Pollen-Coat Protein Bs (PCP-Bs) have been previously found to be required for compatible pollen hydration (Wang et al., 2017) and these PCP-Bs were found to competitively bind ANJ/FER replacing RALF33 to shut down ROS production which would then allow for compatible pollen hydration (Liu et al., 2021a). Pollinating the *anj-1 fer-4* double-mutant stigmas with *pcp-bγ* mutant pollen resulted in increased *pcp-bγ* mutant pollen hydration compared to wild-type stigmas; thus supporting the model that ANJ/FER act in the stigma as negative regulators of pollen hydration (**Figure 1B**; (Liu et al., 2021a).

One would predict from the proposed model by Liu et al., (2021a) that the *pcp-bγ* mutant pollen hydration defect would also be rescued relative to wild-type pollen, but this was not observed suggesting that additional regulators are present in the stigma. Recently, *Arabidopsis* LRR-VIII-2 RLK subgroup members have been identified as positive regulators in the stigma to promote pollen hydration and may be the missing link. RECEPTOR-LIKE KINASE IN FLOWERS 1 (RKF1) and its paralogue, RKF-LIKE 1 (RKFL1) were identified through a yeast two-hybrid screen to interact with a stigma-specific pseudokinase, BRASSIKIN 1 (BKN1; Doucet et al., 2019; Lee and Goring, 2021). These predicted RLKs have extracellular domains composed of a LRR domain followed by a malectin domain (reviewed in Dievart et al., 2020). The *RKF1* and *RKFL1* genes are tandemly linked along with two other paralogs (*RKFL1-RKFL2-RKFL3-RKF1*), and the *RKF1* gene cluster has not been previously ascribed any function. CRISPR-Cas9 generated deletion mutants of the *RKF1* gene cluster (*rkfΔ* mutants) were found to display some phenotypic changes in pollen-stigma interactions (Lee and Goring, 2021). Pollinations of *rkfΔ* mutant pistils with wild-type pollen resulted in significantly slower pollen hydration rates compared to wild-type pistils indicating their role in the stigma as positive regulators of this process. Notably, single *rkf1* and *rkfl1* knockout mutant pistils supported wild-type levels of pollen hydration suggesting functional redundancy within the *RKF1* gene cluster. What remains unknown is what signals from the pollen are being perceived by the extracellular domains of RKF1 and the RKFLs in the stigmatic papillae. Malectin domains in the extracellular regions of CrRLK1Ls perceive RALF peptides for signal transduction (reviewed in Galindo-Trigo et al., 2020b), and ANJ/FER were found to bind PCP-Bs through the second malectin domain in their extracellular domain (Liu et al., 2021a). It is likely that RKF1/RKFLs are binding to other pollen coat signalling peptides to induce the necessary intracellular signals required to initiate water release for pollen hydration (**Figure 1B**).

## Regulators of pollen tube growth in the upper reproductive tract

Immediately following pollen hydration, a pollen tube emerges and grows through the cell wall of the stigmatic papilla to the base of the cell where it continues to grow down between the cells of the stigma and style to reach the transmitting tract. The pollen tube will continue to grow down through the transmitting tract until it senses cues to exit to an unfertilized ovule. RLKs have been identified to function in the reproductive tract to support growth of the compatible pollen tubes. The yeast two-hybrid screen with the stigma-specific BKN1 yielded a second subgroup of RKs, SOMATIC EMBRYOGENESIS RECEPTOR KINASE 1 (SERK1) and BRI1-ASSOCIATED RECEPTOR KINASE 1 (BAK1/SERK3) (Lee and Goring, 2021). While pistils from a *serk1-1 bak1-4* double mutant promoted wild-type pollen hydration, a phenotype was observed for pollen tube growth. Wild-type pollen tubes grew more slowly through the *serk1-1 bak1-4* mutant reproductive tract, failing to grow to the same length as wild-type pollen tubes in wild-type pistils at 6-hours post-pollination. In contrast, there was no change for wild-type pollen tube growth lengths in the *rkfΔ* mutant pistils. However, when the *rkfΔ* mutation was crossed into the *serk1-1 bak1-4* mutant (*serk1-1 bak1-4 rkfΔ*), it exacerbated the phenotype significantly, resulting in further reductions in wild-type pollen tube growth through the mutant reproductive tract at 6 hours post-pollination. Ultimately, some of these pollen tubes failed to reach ovules, as denoted by decreased seed counts in fully developed mutant siliques (Lee and Goring, 2021). Overall, this work indicated that SERK1 and BAK1 in conjunction with the RKF1 cluster play a critical role in the reproductive tract to support pollen tube growth. Ascribing this new biological function to the RKF1 cluster and SERKs is an important step forward in understanding how the pistil sufficiently supports growing compatible pollen tubes (**Figure 1C**).

The SERKs are a subgroup of LRR-RKs with shorter extracellular domains that function as co-receptors with other LRR-RKs in many different processes during plant development and immunity (reviewed in Gou and Li, 2020; Escocard de Azevedo Manhaes et al., 2021). Acting as co-receptors to LRR-RKs, SERKs aid in receiving extracellular signals and transmitting them into the cell for downstream signalling cascades often involving Mitogen Activated Protein Kinases (MAPKs; reviewed in He et al., 2018). Whether SERK1 and BAK1 are forming complexes that include the RKF1/RKFLs in the reproductive tract is not known. However, it is also more likely that additional RKs are involved, given the pattern of SERKs acting as co-receptors for other LRR-RKs. PHYTOSULFOKINE RECEPTOR 1 and 2 (PSKR1 and PSKR2) have been implicated in pollen-pistil interactions (Stuhrwohldt et al., 2015), and these LRR-RKs are known to form complexes with SERK1 and BAK1 in other developmental processes (reviewed in Gou and Li, 2020). Using *pskr1-3 pskr2-1* double mutant pistils, wild-type pollen tubes were observed to grown more slowly through the upper reproductive tract resulting in an increased number of pollen tubes failing to reach an ovule (Stuhrwohldt et al., 2015), a phenotype also observed by Lee and Goring (2021). In addition, Stuhrwohldt et al. (2015) found that phytosulfokine signalling in the pollen tube also promoted pollen tube growth and ovular guidance. Thus, this study implicates phytosulfokine signalling in the reproductive tract to support pollen tube growth, and it would be interesting to see if they are connected to SERK1 and BAK1 in this proposed role (**Figure 1C**).

## Regulators of pollen tube ovular guidance and reception

In order to achieve successful fertilization, the pollen tube needs to successfully exit from the transmitting tract to reach an unfertilized ovule for delivery of the immotile sperm cells for double fertilization of the egg cell and central cell. This is achieved by constant communication between both the male and female gametophytes. The ovule plays an important role in this process since it is responsible for guiding the pollen tube to transversely grow out of the transmitting tract and reach a viable ovule, a process known as ovular guidance. Using both *in-vitro* and semi-*in vivo* experiments with *Torenia fournieri* ovules, it was shown that two cysteine-rich peptides, LURE1 and LURE2, expressed in the synergid cells, were able to attract pollen tubes towards the ovules (Okuda et al., 2009; Goto et al., 2010). This discovery led to the identification of the AtLURE1 peptides secreted by *Arabidopsis* ovules to attract pollen tubes (Takeuchi and Higashiyama, 2012). The pollen tube LRR-RK, POLLEN-SPECIFIC RECEPTOR-LIKE KINASE 6 (PRK6), was then shown to be the key LRR-RK for perceiving AtLURE1 (Takeuchi and Higashiyama, 2016; Zhang et al., 2017). Upon AtLURE1 perception, PRK6 orients the pollen tube tip through re-localization at the plasma membrane and interactions with intracellular signalling proteins such as RopGEFs (guanine nucleotide-exchange factors) for the activation of the ROP1 Rho GTPase (Takeuchi and Higashiyama, 2016). While AtLURE1 has a more species-specific role (Liu et al., 2021b), a second set of related cysteine-rich peptides, XIUQIUs, are also secreted from the synergid cells and play a more non-species-specific function in pollen tube attraction (Zhong et al., 2019). The receptor for perceiving the XIUQIUs is so far unknown (**Figure 1D**).

In the pollen tube, four members of the CrRLK1L family, BUDDHA’S PAPER SEAL (BUPS) 1 and 2, and ANXUR1 and ANXUR2 (ANX1 and 2) are responsible for maintaining pollen tube integrity as the pollen tube grows through the reproductive tract (Miyazaki et al., 2009; Ge et al., 2017; Zhu et al., 2018; reviewed in Hafidh and Honys, 2021; Robichaux and Wallace, 2021). BUPS1/2 form a heterodimeric complex with ANX1/2 to perceive autocrine signals from the pollen tube itself. The signals are secreted RALF4/19 peptides which are detected by the BUPS1/2-ANX1/2 complex to reinforced cell wall integrity and ensure that the pollen tube does not rupture prematurely before arriving at the synergid cells. Pollen tube reception occurs once the pollen tube has reached an ovule and enters the micropyle to come in contact with a receptive synergid cell where the pollen tube will rupture to release the sperm cells. Three additional CrRLK1L family members, FER, ANJ and HERCULES RECEPTOR KINASE 1 (HERK1) are present in the synergid cells for pollen tube reception, where they have a functionally redundant role in promoting pollen tube growth arrest and rupture (Escobar-Restrepo et al., 2007; Galindo-Trigo et al., 2020a) by perceiving several RALFs (RALF 6, 7, 16, 36 and 37) secreted by the pollen tube (**Figure 1E**; Zhong et al., 2022). FER is also proposed to increase ROS production for pollen tube rupture and sperm cell discharge (Duan et al., 2014). Prior to this final stage, FER, ANJ and HERK1 regulate the polytubey block which is proposed to take place at the septum (**Figure 1A**), where additional pollen tubes are prevented from exiting the transmitting tract adjacent to an ovule that has already attracted one pollen tube. FER, ANJ and HERK1 are proposed to carry out this function by perceiving the same pollen tube secreted RALF peptides, RALF 6, 7, 16, 36 and 37 (Zhong et al., 2022).

## Conclusion and future perspectives

The regulation of compatible pollen-pistil interactions leading to fertilization in *Arabidopsis* involves an extensive network of RLKs, RKs and their corresponding ligands. While a number of these players have been identified, there are still many remaining gaps, particularly at the early stages. Starting with the stigmatic papilla regulation of pollen hydration, further work is needed to understand how the negative gating mechanism of the ANJ-FER complex in the papillae connects with the positive regulatory role proposed for the RKF1 cluster. Future investigations should seek to identify ligands for RKF1 and RKFLs as well as potential binding partners in the papilla plasma membrane. Similarly, does the same unknown ligands and binding partners function with RKF1 and the RKFLs in the reproductive tract. As discussed above, SERK1 and SERK3/BAK1 typically form complexes with other LRR-RKs (reviewed in Gou and Li, 2020), and thus future work should focus on identifying which RK complexes are formed in the transmitting tract to support pollen tube growth. Here we have proposed the PSKR1 and PSKR2 as excellent candidates as the *pskr1-3 pskr2-1* double mutant pistils supported displayed slower pollen tube growth through the style (Stuhrwohldt et al., 2015). Finally, downstream signaling pathways activated by these RKs in the reproductive tract will need to be elucidated. The identification of these new RLK players in the reproductive tract is an exciting and important step towards fully understanding this complex process and provides new grounds for additional research into the *Arabidopsis* reproductive processes.

## Author contributions

SB, LC, and DG conceived the review, SB and LC wrote the first draft, SB, LC, and DG edited the review and approved the submitted version.

## Funding

This work was supported by a grant from Natural Sciences and Engineering Research Council of Canada to DG (5010470).

## Acknowledgments

We thank members of the Goring lab for critically reading this article.

## Conflict of interest

The authors declare that the research was conducted in the absence of any commercial or financial relationships that could be construed as a potential conflict of interest.

## Publisher’s note

All claims expressed in this article are solely those of the authors and do not necessarily represent those of their affiliated organizations, or those of the publisher, the editors and the reviewers. Any product that may be evaluated in this article, or claim that may be made by its manufacturer, is not guaranteed or endorsed by the publisher.
